# The Impact of Killer Cell Immunoglobulin-Like Receptors and Human Leukocyte Antigen-E, Human Leukocyte Antigen-G Polymorphisms on Innate Immunity and COVID-19 Severity

**DOI:** 10.1155/jimr/6691437

**Published:** 2025-05-12

**Authors:** Cigdem Kekik, Sonay Temurhan, Yeliz Ogret, Behnoush Nasr Zanjani, Demet Kıvanc, Fatma Savran Oguz, Murat Kose, Fatma Betul Oktelik, Gunnur Deniz

**Affiliations:** ^1^Tissue Typing Laboratory, Department of Medical Biology, Istanbul Faculty of Medicine, Istanbul University, Istanbul, Türkiye; ^2^Institute of Graduate Studies in Health Science, Istanbul University, Istanbul, Türkiye; ^3^Department of Internal Medicine, Istanbul Faculty of Medicine, Istanbul University, Istanbul, Türkiye; ^4^Department of Immunology, Aziz Sancar Institute of Experimental Medicine, Istanbul University, Istanbul, Türkiye

**Keywords:** COVID-19, Human Leukocyte Antigen-E (HLA-E), HLA-G genotype, killer cell immunoglobulin-like receptor (KIR), severe acute respiratory syndrome coronavirus 2 (SARS-CoV-2)

## Abstract

**Background:** Severe acute respiratory syndrome coronavirus 2 (SARS-CoV-2) infection spans a spectrum of symptoms, ranging from mild respiratory issues to severe outcomes like pneumonia, acute respiratory distress syndrome, and fatality. Natural killer (NK) cells, governed by killer cell immunoglobulin-like receptors (KIRs), play a pivotal role in directly combating viral infections. Emerging studies indicate a decline in NK cell numbers and heightened NKG2A expression in infected individuals.

**Objective:** This study focuses on genotyping human leukocyte antigen (HLA)-E, HLA-G, and KIR in SARS-CoV-2-positive individuals, comparing data between those with mild and moderate/severe symptoms. The cohort comprised 100 COVID-19-positive patients and 100 healthy volunteers, both groups subjected to DNA isolation and genotyping using sequence-based sequencing.

**Results:** In 97 COVID-19-positive patients (52 mild, 24 moderate, and 21 severe) and 100 healthy volunteers, the study revealed protective associations with inhibitory alleles (KIR2DL1, KIR2DL3, KIR2DL4, KIR3DL1, KIR3DL2, and pseudo-alleles like KIR3DP1*⁣*^*∗*^003). Conversely, predisposing factors included activator alleles (KIR2DS2, KIR3DS1) and pseudo-alleles (KIR3DP*⁣*^*∗*^001/002). The G^*∗*^01:04 allele and G^*∗*^01:04-G^*∗*^01:04 genotype emerged as protective, while the HLA-E^*∗*^01:03-HLA-E^*∗*^01:03 genotype may negatively impact disease prognosis. Conversely, the HLA-E^*∗*^01:01-HLA-E^*∗*^01:03 and HLA-E^*∗*^01:01-HLA-E^*∗*^01:01 genotypes may confer protection.

**Conclusion:** Genetic variations in KIR, HLA-E, and HLA-G are associated with susceptibility and resistance to severe COVID-19 outcomes. This elucidates the intricate interplay of NK cells and immune-related genes, offering insights into potential therapeutic avenues and personalized approaches.

## 1. Introduction

Coronaviruses (CoVs) are a group of pathogens that emerge periodically, posing significant challenges to human and animal well-being. The latest occurrence is the 2019 Coronavirus Disease (COVID-19) pandemic, underscoring the seriousness of fatalities associated with CoVs [1]. The outbreak of Severe acute respiratory syndrome coronavirus 2 (SARS-CoV-2) infection, initially identified in Wuhan, China, in December 2019, has led to the ongoing COVID-19 pandemic. As of April 2023, it has resulted in a total of 684,904,699 infections and 6,837,598 deaths worldwide [2]. In Turkey, the first case was identified in March 2020, and as of April 2023, there have been 17,232,066 cases and 102,174 deaths [3].

Like in other infections, natural killer (NK) cells assume a pivotal role as the initial defense line in the immune system against COVID-19. They possess the ability to efficiently eradicate the virus and regulate the inflammatory consequences of cytokine storms [4–6]. NK cells are primary effector cells that mediate the natural immune system, and killer cell immunoglobulin-like receptor (KIR) genotypes play a significant role in regulating NK cell activity. The cytotoxic response of NK cells is controlled by a balance between signals from activator or inhibitor receptors on their surfaces. KIRs are encoded by a diverse and variable cluster of genes located within the receptor complex on chromosome 19q13.4, specific to leukocytes. Presently, a total of 14 KIRs have been recognized, which can either induce inhibition (3DL1–3, 2DL1–3, 2DL5), activation (3DS1, 2DS1–5), or both (2DL4) [7]. Research indicates a decline in the overall count of NK cells and a rise in the expression of NKG2A on the surface of NK cells among individuals affected by SARS-CoV-2 infection [8]. In patients undergoing treatment, there is a noted decrease in NKG2A expression alongside a rise in the quantity of NK cells [9]. These findings suggest that SARS-CoV-2 infection could potentially diminish the functionality of NK cells, thereby compromising natural antiviral immunity [7, 10].

Two out of 27 alleles of the nonclassical human leukocyte antigen (HLA) Class I molecule HLA-E (HLA-E^*∗*^01:01, E^*∗*^01:03) have been associated with immune function [11]. Bortolotti et al. demonstrated that the spike 1 protein of SARS-CoV-2 (SP1) increases HLA-E expression. They identified a peptide derived from SP1 that showed high affinity for the HLA-E^*∗*^01:01 allele [12].

Nonclassical HLA-G is considered an important immune checkpoint molecule. Overexpression of HLA-G in viral infections such as COVID-19 facilitates the spread of infection by inhibiting the proliferation of T and B lymphocytes, the activity of antigen-presenting cells (APCs), and CD8+ cytotoxicity. Zhang [13] and colleagues have suggested that the regulation of HLA-G expression in peripheral T cells, B cells, and monocytes may play a role in the replication and clearance of SARS-CoV-2, reflecting a high, low, and high pattern, respectively. This study aims to investigate the potential impact of HLA-E, HLA-G, and KIR genotypes on the severity of COVID-19, providing insights into the role of genetic determinants in immune response, disease progression, and individual susceptibility.

## 2. Methods

### 2.1. Study Population

This case–control study includes 97 individuals diagnosed with COVID-19 and the control group consisted of 100 individuals. Inclusion criteria for the patient group included a laboratory-confirmed SARS-CoV-2 infection via RT-PCR. Exclusion criteria included individuals with known immunodeficiency, chronic inflammatory diseases, or prior COVID-19 vaccination. The control group consisted of individuals with no history of COVID-19, confirmed by a negative antibody test. The sample size was calculated using the online tool available at http://clincalc.com/stats/SampleSize.aspx. To detect a 20% difference between the patient and control groups with a significance level (*α*) of 0.05 and a statistical power of 80%, a minimum of 81 participants per group was required. However, considering potential data losses, we aimed to include 97 individuals in the patient group and 100 individuals in the control group. The study protocol was approved by the Istanbul University Istanbul Faculty of Medicine Ethics Committee, and written consent was obtained from the participants (Approval No: 2021/1393).

Patients with laboratory-confirmed positive results for COVID-19 via real-time polymerase chain reaction (RT-PCR) testing on nasopharyngeal swabs or bronchial aspirates were included. Alleles of KIR genes, as well as allelic and genotypic variations of human leukocyte antigen (HLA)-E and HLA-G, were determined for all patient and control groups, and differences between groups were statistically evaluated.

### 2.2. Genetic Polymorphisms Determination for HLA-E and HLA-G

Peripheral whole blood was obtained from both patients and control subjects, and genomic DNA extraction was carried out from these blood samples using standard methodologies involving the QIAamp DNA Blood Mini Kit (Qiagen, Germany) [14]. DNA purity and concentration were assessed using a NanoDrop spectrophotometer.

### 2.3. Polymerase Chain Reaction and DNA Sequencing

Polymerase chain reaction (PCR) was performed using the following conditions at SensoQuest GmbH (Germany): The procedure commenced with an initial denaturation at 95°C for 10 min, succeeded by 35 cycles of denaturation at 94°C for 45 s, annealing at 60°C for 60 s, extension at 72°C for 1 min, and concluded with a final extension step at 72°C for 10 min. This basic protocol was modified depending on the DNA sample, primer annealing temperatures, and the outcome of amplification. Primers for the analyzed HLA-E and HLA-G genes were obtained from Synbio Technologies (USA). PCR products were visualized on a 1%–2% agarose gel with ethidium bromide for the assessment of successful results [15]. The HLA-E genotyping could not be performed for two of the patients included in the study.

### 2.4. Enzymatic Purification of PCR Products and DNA Sequencing

PCR products underwent purification utilizing ExoSAP-IT (Applied Biosystems) following the guidelines provided by the manufacturer. DNA sequencing was performed using an ABI 3730XL DNA sequencer (Applied Biosystems) and BigDye 3.1 (Applied Biosystems). This method utilizes fluorescent dyes attached to terminating dideoxynucleotide triphosphates (ddNTPs), and DNA fragments were electrophoretically defined. Each PCR product was displayed in separate reactions using forward and reverse primers. Sequence chromatograms were visualized using Sequencing Analysis version 5.3.1 (ABI) and analyzed by aligning them with the BLAST website at the National Center for Biotechnology Information [16].

### 2.5. Determination of Genetic Polymorphisms of KIRs

KIR genotyping was conducted using the Texas-Biogen SBT KIR Genotyping Kit recommended by the manufacturer, and all steps were followed meticulously. The thermal cycling steps included an initial heating phase at 95°C for 1 min, followed by 30 cycles comprising 20 s at 94°C, 20 s at 63°C, and 90 s at 72°C. The kit encompasses alleles for 2DL1, 2DL2, 2DL3, 2DL4, 3DL1, 3DL2, 3DL3, 2DS2, 3DS1, 2DP1, 3DP1*⁣*^*∗*^001/002, and 3DP1*⁣*^*∗*^003 [17]. In the KIR genotyping analysis, results could not be obtained for 23 patients and 12 controls despite repeated attempts, and it was not possible to obtain new samples.

### 2.6. Statistical Analysis

Statistical analyses were conducted using IBM SPSS version 29.0.0.0. Descriptive statistics were reported as mean ± standard deviation for continuous variables and frequency (%) for categorical variables. Group comparisons were performed using the Chi-square test or Fisher's exact test for categorical data, and independent *t*-tests or Mann–Whitney *U* tests for continuous variables, depending on data distribution. A *p*-value of < 0.05 was considered statistically significant. To adjust for multiple comparisons, Bonferroni correction was applied where necessary. Graphics of the data were performed using the R programing language (version 4.3.2). Genotyping analysis could not be performed for two patients in the HLA-E group and for 23 patients and 12 controls in the KIR genotyping group. These individuals were excluded from the respective statistical analyses, and sensitivity analyses were conducted to assess the potential impact of missing data.

## 3. Results

A total of 97 patients (M/F: 52/45, mean age: 52.78 ± 14.08) and 100 healthy individuals (M/F: 42/58, mean age: 54.2 ± 6.82) were included in our study. Patients were categorized as mild (*n* = 52), moderate (*n* = 24), and severe (*n* = 21) based on clinical findings. The age and gender distributions of the groups are presented in Table 1. There was no statistical significance among the comparison of COVID-19 patients, subgroups, and controls based on gender (p  > 0.05).

**Figure 1:**
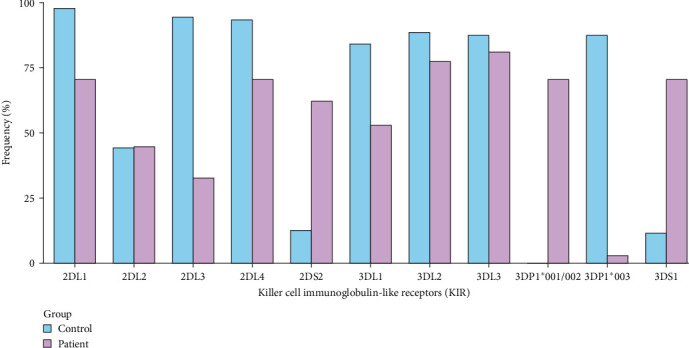
KIR allele frequencies in control and patient groups. The figure illustrates the frequencies of KIR in control and patient groups. The data were processed and visualized by the authors using R programing. The bars represent the percentage frequencies of different KIR receptors observed in both control and patient groups. KIR, killer cell immunoglobulin-like receptor.

When comparing KIR alleles between the patient and control groups, 2DL1, 2DL3, 2DL4, 3DL1, 3DL2, and 3DP1*⁣*^*∗*^003 were statistically decreased, whereas 2DS2, 3DS1, and 3DP1*⁣*^*∗*^001/002 were significantly increased in the patient group. The bar plot presented in Figure 1 illustrates the percentage frequencies of specific KIR alleles in the control and patient groups. The frequencies are represented on the *y*-axis as a percentage, while the KIR alleles are depicted on the *x*-axis.

In individuals experiencing mild illness, 2DL2 and 3DL3 were statistically lower compared to controls. There was no statistically significant difference in 3DL1 and 3DL2 alleles between severe cases and controls (as opposed to those with mild and moderate severity). The comparison of KIR alleles between the control group and patients experiencing the disease with mild, moderate, or severe severity is provided with *p* values in Table 2.

**Figure 2:**
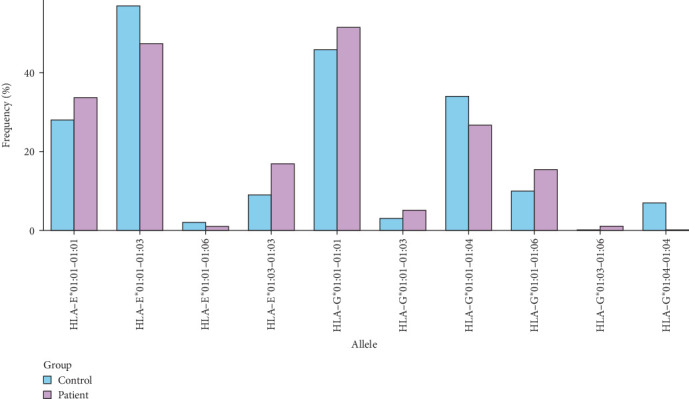
HLA allele frequencies in control and patient groups. The bars represent the frequencies of different alleles observed in both control and patient groups. (All components of this figure were generated by the authors using R programing unless otherwise stated). HLA, human leukocyte antigen.

Our investigation into the diversity of HLA alleles revealed distinct patterns in both control and patient groups. HLA alleles play a crucial role in immune response modulation, and variations in their frequencies can have implications for disease susceptibility and progression.  Figure 2 provides a visual representation of the percentage frequencies of specific HLA alleles in both the control and patient groups. The *x*-axis denotes the HLA alleles, while the *y*-axis represents the frequency percentage.

No statistical significance was observed in terms of HLA-E alleles between the patient and control groups. The E^*∗*^01:01 allele exhibited an elevated presence in mild cases but a diminished one in severe cases compared to controls (*p* < 0.001). Conversely, the E^*∗*^01:03 allele demonstrated a decreased frequency in mild cases but an increased one in severe cases when compared to controls (*p* < 0.002) (Table 3). In contrast, when compared with the control group, the G^*∗*^01:04 allele demonstrated statistically lower frequencies both in the entire patient cohort and specifically in those with mild severity (Table 3). HLA-E^*∗*^01:01 homozygosity was prevalent in mild cases (*p* < 0.001) but was statistically less frequent in severe cases (*p* < 0.042). Remarkably, severe cases exhibited a notably higher prevalence of HLA-E^*∗*^01:03 homozygosity (Table 4). In the control group, HLA-G^*∗*^01 : 04 homozygosity exhibited statistical significance compared to all patients (*p* < 0.014). Furthermore, while HLA-G^*∗*^01:01 homozygosity was prominent in mild cases, HLA-G^*∗*^01:01–01:04 heterozygosity was notably higher in the control group (Table 4).

## 4. Discussion

SARS-CoV-2, responsible for coronavirus disease 19 (COVID-19), affects the respiratory system, resulting in changes in the severity of lung abnormalities. This is marked by decreased gas exchange due to alveolar and interstitial edema [18]. COVID-19 primarily targets the respiratory system, facilitated by interactions between viral components and the immune system, including NK cells and HLAs. NK cells, crucial in the early immune response, and KIRs, which regulate these cells, play vital roles in managing the infection's severity. The expression of HLA-I molecules, essential for the control of both natural and acquired immune system components, is thought to potentially influence susceptibility or resistance to COVID-19 [19–21].

KIRs are encoded by a highly polymorphic set of genes located within the leukocyte receptor complex on chromosome 19q13.4. Currently, 14 KIRs have been identified that trigger either inhibition (3DL1–3, 2DL1–3, 2DL5), activation (3DS1, 2DS1–5), or both (2DL4) [7]. Inhibitory receptors play a critical role in tolerance. Binding to self HLA class I ligands sends inhibitory signals that prevent cytolysis. However, when viral infection downregulates these HLA class I ligands, these inhibitory signals are blocked, leading to the destruction of the target cells [22, 23]. In a study conducted by Hajeer et al. [24], there is evidence of a relationship between the activating feature KIR2DS4 and the inhibitory feature KIR3DL1, indicating an increased risk of severity in COVID-19 disease. The study illustrated that KIR2DS4 is uniquely linked to severe cases of COVID-19, whereas both KIR2DS4 and KIR3DL1 are associated with mild, moderate, and severe manifestations of the disease [25]. Despite both genes being prevalent in the general population according to previous studies, KIR2DS4 and KIR3DL1 seem to contribute to susceptibility to the severity of COVID-19 disease [24, 25]. In a study published by Farias et al. in 2024, it was reported that KIR2DS4001 allele weakened the protective effect against disease [26]. In our study, KIR2DS4 was not detected, while KIR2DL1 was associated with a potential protective effect against the disease. However, further research with larger and more diverse cohorts is needed to confirm this association. In a study examining the immunogenetics of COVID-19, a positive correlation was discovered between KIR2DL3 and daily mortality rates, while an inverse association was observed between activating KIR2DS2 and daily mortality rates. These findings are consistent with the complex role of KIR genotypes in regulating immune responses. A study on the immunogenetics of COVID-19 reported a positive correlation between KIR2DL3 and daily mortality rates, suggesting that some inhibitory KIR alleles may compromise immune defense. Conversely, an inverse association was identified between the activating KIR2DS2 and daily mortality rates, indicating that activating alleles can also contribute to favorable outcomes under specific conditions. The same study emphasized the variability in the effects of inhibitory KIR genotypes and their interactions with HLA molecules on COVID-19 outcomes. While certain inhibitory signals, such as those mediated by KIR2DL1, appear to offer protection, others, like KIR2DL3, may hinder effective immune responses [27]. Parikh et al. [28] further highlighted the importance of inhibitory NK cell receptors, demonstrating that MHC-I-restricted, inhibitory NK cell receptor-dependent antiviral effects, such as those mediated by Ly49 receptors, play a crucial role in clearing viral infections like MCMV. These findings suggest a broader role for NK cell activation receptors in shaping immune defense mechanisms, which may provide valuable insights into the regulation of immune responses in diseases such as COVID-19 [28]. In our study, inhibitory alleles such as KIR2DL1, KIR2DL4, KIR3DL1, and KIR3DL2, as well as the pseudo-allele KIR3DP1*⁣*^*∗*^003, were associated with a potential protective effect against the disease. In contrast, activating alleles such as KIR2DS2 and KIR3DS1, along with the pseudo-alleles KIR3DP1*⁣*^*∗*^001/002 and the inhibitory allele KIR2DL3, were linked to increased susceptibility. However, further studies with larger sample sizes are required to validate these associations.

The main role of HLA-G is to regulate the immune system by engaging with receptors such as ILT-2, ILT-4, and KIR2DL4 [29]. Çelik et al. [30] highlighted the significant role of HLA-G genetic variation in immune regulation, demonstrating that a single amino acid difference in two domains of HLA-G can influence NK cell-mediated lysis. Their findings revealed that the HLA-G^*∗*^01:04 allele possesses a stronger immune-suppressive function compared to the HLA-G^*∗*^01:01 and HLA-G^*∗*^01:03 alleles, likely due to its stronger affinity for the inhibitory NKG2A/CD94 receptor complex [31]. This suggests a mechanism by which the HLA-G^*∗*^01:04 allele could modulate immune responses, particularly in the context of diseases such as COVID-19, where NKG2A expression is closely linked to disease severity [8]. In our study, the HLA-G^*∗*^01:04 allele and the G^*∗*^01:04-G^*∗*^01:04 genotype were associated with a potential protective effect, a finding that aligns with and builds upon the observations of Çelik et al. However, further research is needed to validate this association in larger and more diverse populations. While their study emphasized the immune-suppressive capacity of the HLA-G^*∗*^01:04 allele, our results suggest that this allele may confer protection against severe disease progression. This dual functionality of HLA-G underscores its complex role in immunity, acting as both a suppressor in infections and tumors and a protector in specific contexts, such as immune-privileged sites. The interplay between HLA-G^*∗*^01:04 and NK cell function highlights its critical involvement in immune regulation. The suppressive properties of HLA-G^*∗*^01:04, mediated through its interaction with NKG2A/CD94, may reduce excessive immune activation, thus offering protection in certain pathological conditions. Conversely, in chronic infections or severe diseases, this immune suppression could compromise effective viral clearance. The findings from both studies underscore the importance of understanding the context-dependent role of HLA-G in modulating immune responses, with significant implications for its potential as a biomarker for disease susceptibility and as a target for therapeutic interventions in infections such as SARS-CoV-2.

HLA-G could modulate the expression of HLA-E, particularly through the different isoforms of HLA-G that can produce various types of peptides binding to the HLA-E pocket, influencing its surface expression. Moreover, in diverse physiological and pathological contexts, HLA-G and HLA-E are naturally coexpressed in various cell populations, forming an environment that suppresses the immune response [32]. The heightened expression of both HLA-E and NKG2A/CD94 on infected cells and NK cells instigates the suppression of NK cell cytotoxicity, secretion, and degranulation [33, 34]. In vivo examinations have revealed a rise in circulating NKG2A/CD94-positive lymphocytes (comprising both NK and T cells) in COVID-19 patients, accompanied by elevated IFN-*γ* secretion. This is accompanied by a decrease in cytotoxicity, dropping from 80% to 16% compared to control groups. The interaction between HLA-E and NKG2A provides a partial explanation for the noted anergic condition observed in severe COVID-19 patients, ultimately resulting in the depletion of NK cells [35, 36]. In research conducted by Vietzen et al., it was found that individuals with severe COVID-19 exhibit a higher prevalence of both homozygous and heterozygous HLA-E^*∗*^01:01 alleles in comparison to those with milder forms of the disease. Additionally, the S1 peptide exhibits higher affinity for HLA-E^*∗*^01:01 than HLA-E^*∗*^01:03, supporting the role of the HLA-E/S1 peptide complex in generating NK cell anergy and exacerbating the severity of COVID-19 [37].

HLA-E is characterized by a complex molecular structure that is associated with a distinct subset of peptides derived from the leader sequences of MHC class I molecules. This intricate configuration facilitates the efficient transport of these peptides to the cell surface, ensuring their stability for optimal interaction with NK cell receptors [38]. Most viruses, including SARS-CoV-2, evade recognition and cytotoxicity by the cellular immune system through cytotoxic T cells by reducing MHC class I expression. Various polymorphisms of HLA-E have been identified; among them, the two functional alleles, HLA-E^*∗*^01:01 and HLA-E^*∗*^01:03, and their three genotypes HLA-E^*∗*^01:01-HLA-E^*∗*^01:01, HLA-E^*∗*^01:03-HLA-E^*∗*^01:03, and HLA-E^*∗*^01:01-HLA-E^*∗*^01:03 hold clinical significance. Studies have additionally demonstrated that the HLA-E molecule derived from the HLA-E^*∗*^01:03 allele exhibits higher cell surface expression and greater stability compared to HLA-E^*∗*^01:01. This variation could lead to a more effective inhibitory role upon binding with the NKG2A receptor, consequently eliciting distinct responses from NK cells to each of these ligands [39]. Considering the pivotal role of HLA-E as a major modulator of NK cell function and its depletion in the pathogenesis of COVID-19, this investigation identifies an association between HLA-E polymorphism and susceptibility to SARS-CoV-2 [34]. Certain viruses can activate NK cells by downregulating ligands on infected cells that normally inhibit NK cell activity via interactions with inhibitory receptors, such as KIRs and the C-type lectin-like receptor CD94-NKG2A [40]. The reduction in MHC class I expression during viral infections further diminishes HLA-E levels, as MHC class I molecules provide the peptides necessary for optimal HLA-E expression and its interaction with NKG2A. Hosseini [39] et al. reported that individuals carrying the HLA-E^*∗*^01:03-HLA-E^*∗*^01:03 genotype have a higher likelihood of experiencing severe disease. Their study indicates that HLA-E molecules presenting HIV-derived epitopes can increase the susceptibility of target cells to NK cell-mediated lysis during the early stages of HIV infection. However, during chronic infection, sustained elevated expression of HLA-E may lead to NK cell dysfunction, potentially undermining effective viral control. Similarly, our study revealed a higher prevalence of the HLA-E^*∗*^01:03-HLA-E^*∗*^01:03 genotype among patients with severe disease progression. Additionally, the HLA-E^*∗*^01:01-HLA-E^*∗*^01:03 genotype was more frequently observed in the control group compared to the severe patient group, while the HLA-E^*∗*^01:01-HLA-E^*∗*^01:01 genotype was more prevalent in both the overall patient cohort and the severe patient subgroup compared to the control group. These findings contribute to a better understanding of the potential role of HLA-E genotypes in disease progression and severity. However, further studies are required to confirm these associations in larger and more diverse populations.

In conclusion, our study sheds light on the intricate interplay between host genetic factors and the severity of COVID-19 caused by SARS-CoV-2. Our investigation focused on the role of HLA-I molecules and KIRs in influencing susceptibility or resistance to COVID-19. We explored the prevalence of different HLA alleles (-E -G) and KIRs in a Turkish population, revealing potential associations with increased vulnerability or protection against COVID-19. Notably, KIRs, encoded by polymorphic genes on chromosome 19q13.4, demonstrated their crucial role in either inhibiting or activating immune responses. Moreover, the study delved into the immunomodulatory function of HLA-G and its interaction with HLA-E, revealing significant associations with COVID-19 severity. The intricate relationship between HLA-E polymorphisms and NK cell responses was explored, providing insights into the observed anergic state in severe COVID-19 patients. In summary, our study contributes valuable insights into the complex genetic landscape of COVID-19 susceptibility and severity in the Turkish population. Understanding these genetic associations is crucial for personalized risk assessment, treatment strategies, and further research into the host–virus interaction dynamics.

## Figures and Tables

**Table 1:** Distribution of patients and controls based on gender and age.

Severity of disease	Gender		Total patients
	Female	Male	
	—	—	—
Mild (*n*)	26	26	52
Mean age (SD)	44.42 (10.29)	48.58 (14.45)	46.5 (12.59)
	—	—	—
Moderate (*n*)	10	14	24
Mean age (SD)	54.8 (13.12)	58.5 (10.74)	57 (11.33)
	—	—	—
Severe (*n*)	9	12	21
Mean age (SD)	63 (12.48)	63.31 (12.57)	63.18 (12.23)
	—	—	—
Total patients (*n*)	45	52	97
Mean age (SD)	50.44 (13.48)	54.81 (14.4)	52.78 (14.08)
	—	—	—
Control (*n*)	58	42	100
Mean age (SD)	53.6 (6.6)	55.7 (7.1)	54.2 (6.82)

Abbreviations: *n*, number; SD, standard deviation.

**Table 2:** Comparison of KIR allele frequencies in COVID-19 patients and subgroups with controls.

Gene	Allele	Control (*n* = 176)	Patient (*n* = 148)		Mild (*n* = 58)		Moderate (*n* = 48)		Severe (*n* = 42)	
KIR		*n* (%)	*n* (%)	*p* Value	*n* (%)	*p* Value	*n* (%)	*p* Value	*n* (%)	*p* Value
	2DL1	172 (97.7)	104 (70.27)	*p* < 0.001 OR = 0.053 CI = 0.018–0.15	30 (51.72)	*p* < 0.001 OR = 0.025 CI = 0.008–0.076	34 (70.83)	*p* < 0.001 OR = 0.056 CI = 0.018–0.182	40 (95.24)	*p*=0.031 OR = 0.23 CI = 0.056–0.97

	2DL2	78 (44.3)	66 (44.6)	*p* > 0.05	16 (27.59)	*p*=0.024 OR = 0.479 CI = 0.25–0.915	26 (54.17)	*p* > 0.05	24 (57.14)	*p* > 0.05

	2DL3	166 (94.3)	48 (32.43)	*p* < 0.001 OR = 0.028 CI = 0.014–0.059	18 (13.79)	*p* < 0.001 OR = 0.010 CI = 0.004–0.026	38 (79.17)	*p*=0.001 OR = 0.229 CI = 0.089–0.589	2 (4.76)	*p* < 0.001 OR = 0.003 CI = 0.001–0.014

	2DL4	164 (93.2)	104 (70.27)	*p* < 0.001 OR = 0.169 CI = 0.085–0.335	24(41.38)	*p* < 0.001 OR = 0.053 CI = 0.024–0.117	44 (91.67)	*p* > 0.05	36 (85.71)	*p*=0.019 OR = 0.329 CI = 0.125–0.864

	3DL1	148 (84.1)	78 (52.7)	*p* < 0.001 OR = 0.205 CI = 0.122–0.343	34 (58.62)	*p* < 0.001 OR = 0.268 CI = 0.138–0.519	10 (20.83)	*p* < 0.001 OR = 0.05 CI = 0.022–0.11	34 (80.95)	*p* > 0.05

	3DL2	156 (88.6)	114 (77.03)	*p*=0.004 OR = 0.42 CI = 0.23–0.76	38 (65.52)	*p* < 0.001 OR = 0.244 CI = 0.119–0.497	42 (87.5)	*p* > 0.05	34 (80.95)	*p* > 0.05

	3DL3	154 (87.5)	120 (81.08)	*p* > 0.05	40 (68.97)	*p*=0.005 OR = 0.357 CI = 0.172–0.743	44 (91.67)	*p* > 0.05	36 (85.71)	*p* > 0.05

	2DS2	22 (12.5)	92 (62.16)	*p* < 0.001 OR = 11.3 CI = 6.842–19.69	26 (46.83)	*p* < 0.001 OR = 5.87 CI = 2.96–11.66	32 (66.67)	*p* < 0.001 OR = 14 CI = 6.63–29.58	34 (80.95)	*p* < 0.001 OR = 23.8 CI = 10.33–54.84

	3DS1	20 (11.4)	104 (70.27)	*p* < 0.001 OR = 18.44 CI = 10.28–33.06	26 (46.83)	*p* < 0.001 OR = 6.54 CI = 3.25–13.15	46 (95.83)	*p* < 0.001 OR = 179.4 CI = 40.42–796.2	32 (76.19)	*p* < 0.001 OR = 22.69 CI = 9.91–51.95

	3D*P*1*⁣*^*∗*^001/002	0 (0)	118 (79.73)	*p* < 0.0001 OR = 1,468 CI = 89–24,291	36 (62.07)	*p* < 0.0001 OR = 629 CI = 37–10,636	48 (100)	*p* < 0.0001 OR = 34,241 CI = 670.1–1,749,672	34 (80.95)	*p*=0.0001 OR = 1,432 CI = 80.7–25,425

	3D*P*1*⁣*^*∗*^003	154 (87.5)	4 (2.7)	*p* < 0.001 OR = 0.004 CI = 0.001–0.012	4 (6.9)	*p* < 0.001 OR = 0.011 CI = 0.03–0.032	0 (0)	*p* < 0.0001 OR = 0.001 CI = 0.00008–0.002	0 (0)	*p*=0.0001 OR = 0.001 CI = 0.00009–0.002

Abbreviations: CI, confidence interval; KIR, killer cell immunoglobulin-like receptor; *n*, number; OR, odds ratio; *p*, *p* value.

**Table 3:** Comparison of HLA-E and HLA-G alleles in COVID-19 patients and subgroups with controls.

Gene	Allele	Control (*n* = 200)	Patient (*n* = 190)		Mild (*n* = 104)		Moderate (*n* = 46)		Severe (*n* = 40)	
HLA-E		*n* (%)	*n* (%)	*p* Value	*n* (%)	*p* Value	*n* (%)	*p* Value	*n* (%)	*p* Value
	01:01	116 (58)	110 (57.9)	*p* > 0.05	80 (74.9)	*p*=0.001 OR = 2.41 CI = 1.41–4.12	23 (50)	*p* > 0.05	7 (17.5)	*p* < 0.001 OR = 0.154 CI = 0.065–0.364

	01:03	81 (40.5)	79 (41.6)	*p* > 0.05	24 (23.1)	*p*=0.002 OR = 0.441 CI = 0.258–0.754	22 (47.8)	*p* > 0.05	33 (82.5)	*p* < 0.001 OR = 6.93 CI = 2.922–16.42

	01:06	3 (1.5)	1 (0.5)	*p* > 0.05	0 (0)	*p* > 0.05	1 (2.2)	*p* > 0.05	0 (0)	*p* > 0.05

**Gene**	**Allele**	**Control (*n* = 200)**	**Patient (*n* = 194)**		**Mild (*n* = 104)**		**Moderate (*n* = 48)**		**Severe (*n* = 42)**	
**HLA-G**		***n* (%)**	***n* (%)**	***p* Value**	***n* (%)**	***p* Value**	***N* (%)**	***p* Value**	***n* (%)**	***p* Value**

	01:01	139 (69.5)	146 (75.3)	*p* > 0.05	83 (79.8)	*p* > 0.05	34 (70.8)	*p* > 0.05	29 (69)	*p* > 0.05

	01:03	3 (1.5)	6 (3.1)	*p* > 0.05	6 (5.8)	*p* > 0.05	0 (0)	*p* > 0.05	0 (0)	*p* > 0.05

	01:04	48 (24)	27 (13.9)	*p*=0.011 OR = 0.512 CI = 0.304–0.861	7 (6.7)	*p* < 0.001 OR = 0.229 CI = 0.099–0.526	9 (18.8)	*p* > 0.05	11 (26.2)	*p* > 0.05

	01:06	10 (5)	15 (7.7)	*p* > 0.05	8 (7.7)	*p* > 0.05	5 (10.4)	*p* > 0.05	2 (4.8)	*p* > 0.05

Abbreviations: CI, confidence interval; HLA, human leukocyte antigen; *n*, number; OR, odds ratio; *p*, *p* value.

**Table 4:** Comparison of HLA-E and HLA-G genotypes in COVID-19 patients and subgroups with controls.

Genotype	Control (*n* = 100)	Patient (*n* = 95)		Mild (*n* = 52)		Moderate (*n* = 23)		Severe (*n* = 20)	
HLA-E^*⁣*^*∗*^^	*n* (%)	*n* (%)	*p* Value	*n* (%)	*p* Value	*n* (%)	*p* Value	*n* (%)	*p* Value
01:01–01 : 01	28 (28)	32 (33.7)	*p* > 0.05	29 (55.8)	*p*=0.001 OR = 3.242 CI = 1.65–6.529	2 (8.7)	*p* > 0.05	1 (5)	*p*=0.042 OR = 0.135 CI = 0.017–1.059

01:01–01:03	57 (57)	45 (47.4)	*p* > 0.05	22 (42.3)	*p* > 0.05	18 (78.3)	*p* > 0.05	5 (25)	*p*=0.009 OR = 0.251 CI = 0.085–0.746

01:03–01:03	9 (9)	17 (17.9)	*p* > 0.05	1 (1.9)	*p* > 0.05	2 (8.7)	*p* > 0.05	14 (70)	*p* < 0.001 OR = 23.593 CI = 7.276–76.415

01:01–01:06	2 (2)	1 (1.05)	*p* > 0.05	0	*p* > 0.05	1 (4.3)	*p* > 0.05	0 (0)	*p* > 0.05

**Genotype**	**Control (*n* = 100)**	**Patient (*n* = 97)**		**Mild (*n* = 52)**		**Moderate (*n* = 24)**		**Severe (*n* = 21)**	
**HLA-**G^*⁣*^*∗*^^	***n* (%)**	***n* (%)**	***p* Value**	***n* (%)**	***p* Value**	***n* (%)**	***p* Value**	***n* (%)**	***p* Value**

01:01–01:01	46 (46)	50 (51.5)	*p* > 0.05	32 (61.54)	*p*=0.001 OR = 3.242 CI = 1.65–6.529	10 (41.7)	*p* > 0.05	8 (38.1)	*p* > 0.05

01:01–01:03	3 (3)	5 (5.15)	*p* > 0.05	5 (9.62)	*p* > 0.05	0 (0)	*p* > 0.05	0 (0)	*p* > 0.05

01:01–01:04	34 (34)	26 (26.8)	*p* > 0.05	6 (11.54)	*p*=0.003 OR = 0.253 CI = 0.098–0.652	9 (37.5)	*p* > 0.05	11 (52.38)	*p* > 0.05

01:01–01:06	10 (10)	15 (15.46)	*p* > 0.05	8 (15.38)	*p* > 0.05	5 (20.83)	*p* > 0.05	2 (9.52)	*p* > 0.05

01:04–01:04	7 (7)	0 (0)	*p*=0.014 OR = 0.06 CI = 0.003–1.136	0 (0)	*p* > 0.05	0 (0)	*p* > 0.05	0 (0)	*p* > 0.05

01:03–01:06	0 (0)	1 (1.03)	*p* > 0.05	1 (1.92)	*p* > 0.05	0 (0)	*p* > 0.05	0 (0)	*p* > 0.05

Abbreviations: CI, confidence interval; HLA, human leukocyte antigen; *n*, number; OR, odds ratio; *p*, *p* value.

## Data Availability

All data and material are organized into databases and available from the corresponding author upon request.
